# A Clinical Review of the Association of Thyroid Stimulating Hormone and Cognitive Impairment

**DOI:** 10.1155/2013/856017

**Published:** 2013-09-23

**Authors:** Sylvia Annerbo, Johan Lökk

**Affiliations:** ^1^Division of Clinical Geriatrics, Department of Neurobiology, Care Sciences and Society, Karolinska Institutet, Stockholm, Sweden; ^2^Geriatric Department, Karolinska University Hospital Huddinge, Stockholm, Sweden

## Abstract

Clinical and subclinical hypothyroidism as well as overt hyperthyroidism in middle-aged and elderly adults are both associated with decreased cognitive functioning as memory, reaction time, and visuospatial organization. Subclinical hyperthyroidism (SH) or low serum concentrations of TSH concentrations have been associated with dementia in previous epidemiological studies, but the association in the elderly has not been established. There is little or no consensus regarding how thyroid function is associated with cognitive performance in the elderly. In this focused review, we have performed an examination between eleven studies from the last five years examining the association between thyroid function and cognitive performance in elderly people, a group who is overrepresented among individuals with minor abnormalities in serum TSH and thyroid hormone concentration. Six of the studies showed a consistent finding of an association between SH with cognitive impairment or dementia. In general, taking into account the largest and most powerfully designed studies, there is a strong body of evidence supporting the association between SH and cognitive impairment. The scarce number of publications on these topics indicates the need of more research especially regarding longitudinal and interventional studies thus hopefully enabling confirmation or rejection of causality between TSH abnormalities and dementia.

## 1. Background

Dementia is a clinical syndrome that is characterized by progressive loss of cognitive capabilities and functional impairment. The population prevalence of dementia is about 1% in individuals aged 60–64 years and increases up to 45% in the most advanced ages of 95 and above [1]. Cognitive performance among older people is complex and dependent of many factors, and in spite of the known effects of clinical thyroid disorders on cognitive function, little is known about the relationship between thyroid hormone (TH) levels and cognitive performance among older people with levels of TH within the normal reference range. Since thyroid hormone concentrations change with age and since cognitive decline is often concomitant with aging, physiological changes in thyroid function might be casually related to changes in cognition during normal aging.

A lot of studies have investigated the association between dementia, particularly Alzheimer's disease (AD), and thyroid function, but the findings are inconsistent [2–9].

Subclinical hyperthyroidism (SH) is a term used to define a clinical condition with reduced serum thyroid stimulating hormone (TSH) level while free thyroxine (FT4) and tri-iodothyronine (FT3) levels stay within the reference ranges. The cause is either exogenous thyroid hormone therapy or endogenous overproduction of FT4 and/or FT3. Approximately 1-2% of patients over the age of 60 with SH progress to overt hyperthyroidism. Most people with SH have no symptoms of hyperthyroidism. A small number of individuals with SH have early the Graves disease, and in these cases progression to overt hyperthyroidism is more probable [10]. On the other hand, 1-2% of elderly individuals have a constant and stable low serum TSH that remains unexplained by any primary thyroid pathology. SH is also associated with an increased risk of developing cardiovascular complications as atrial fibrillation and coronary heart disease, osteoporosis, and dementia [11, 12].

Subclinical thyroid diseases, with variations in thyroid function within the normal range and elevated or suppressed TSH levels with normal levels of FT3 and FT4, have often been connected with cognitive dysfunction [13, 14].

It is unclear why high/normal FT4 levels are independently associated with cognitive decline in people without clinical disease. Subclinical as well as clinical thyroid diseases are shown to be connected with cardiovascular disease and vascular risk factors with accumulating epidemiologic evidence that vascular risk factors increase the risk of AD, indicating a connection between thyroid function and AD [2, 15–17]. 

The *β* amyloid plaque is the pathological characteristic of AD, and the potential pathways of *β* amyloid-mediated neurotoxicity include inhibition of acetylcholine activity in the cortex and hippocampus, oxidative stress from free radical damage, mitochondrial dysfunction, and apoptotic cell death [18, 19]. There has also been shown that thyroid function influences systemic oxidative stress [20]. There is evidence supporting an interrelationship between thyroid hormone (TH) and the cholinergic system, which is affected early in AD [21]. Furthermore, TH negatively regulates the expression of the amyloid-*β* protein precursor, which has an essential role in AD development. It is postoperatively commonly occurring with euthyroid sick syndrome characterized by reduced T3 and T4 levels, maybe as a consequence of the psychophysical stress of surgery. This phenomenon often coincides with a transient reversible alteration in cognition in elderly patients indicating the impact of TH on cognition. TH can be metabolized in peripheral tissue by deiodination, conjugation, deamination, and decarboxylation enzyme reactions [22]. Thus alterations in these metabolic pathways might impact the quantity of TH metabolites influencing function at a cellular level. 

It is unclear whether thyroid dysfunction results from or contributes to AD pathology. The hippocampus and nucleus of amygdala in the medial temporal lobe have a high density of thyroid hormone receptors, and patients with mild AD have more atrophy in those parts of the brain compared to healthy elderly [23]. 

There are a number of possible explanations for the finding of lowered TSH in AD. A decrease of the thyrotrophic-releasing hormone (TRH) can enhance the phosphorylation of tau-protein and other proteins that are potentially involved in the pathogenesis of AD [24]. TRH increases the acetylcholine synthesis and release in rats, indicating that reduction of TRH may cause acetylcholine decrease, an important factor in the development of AD [25]. 

In this focused review paper, we wanted to concentrate on the question whether TSH has an impact on development of AD. 

## 2. Method

We searched for papers on the Pub Med, Embase electronic database using the terms “TSH, subclinical thyroid disorders, subclinical hyperthyroidism, dementia, cognitive impairment, dementia, and Alzheimer's disease” and found only eleven papers matching the search terms during the last five years. The studies included in the review evaluated a total number of 22, 210 patients. Five of the studies (with 2 181 objects) found no association between low serum TSH and cognitive decline, whereas six studies (with 20 030 objects) showed a connection.

 Six of the studies had a prospective longitudinal population-based design, and five were cross-sectional/case-control studies.

## 3. Results

### 3.1. Relationship between SH and Cognitive Decline

*Longitudinal Population-Based Studies (Table 1)*. The largest population-based study from the least years involved 12 115 participants with a mean age of 66.5 years and a median follow-up period of 5.6 years. The study included 2 004 persons with SH and 10 111 euthyroid. Of the SH patients, 1 491 subjects had serum TSH concentrations from 0.1 to 0.4 mU/L (group 1), and 414 subjects TSH had concentrations below 0.1 mU/L (group 2). A positive association between SH and dementia was observed (adjusted hazard ratio (HR), 1.64; 95% confidence interval (CI), 1.2–2.25). Also, for group 1 a significant association was observed (adjusted HR, 1.77; 95% CI, 1.24–2.52), whereas for group 2 no relationship was shown [25].

 In the Honolulu Heart Program, de Jong et al. carried out a study with 615 men, with a mean age of 77.5 years and a mean follow-up period of 4.7 years [26]. No association was shown between TSH and the risk of dementia. However, for FT4 a 20% increased risk for dementia and a 30% increased risk for AD for each standard deviation increase in serum FT4 (HR for dementia, 1.21; 95% CI, 1.04–1.40; and HR for AD, 1.31; 95% CI, 1.14–1.51). 

Positive findings were also shown in a large population-based study with 1864 participants with a mean age of 71 years and a follow-up period of 12.7 years [27]. In this community-based observational study, the patients were divided into three sex-specific tertiles according to baseline TSH: T1, less than 1.0 mU/L; T2, 1.0–2.1 mU/L, and T3 more than 2.1 mU/L. Both low and high TSH were associated with an increased risk of developing incident AD in women but not in men, in the lowest tertile (HR, 2.26; 95% CI, 1.36–3.77; *P* = 0.002) and in the highest (HR, 1.84; 95% CI, 1.10–3.08; *P* = 0.003).

 In “The Health in Men study,” Yeap et al. carried out a study with 3401 community-dwelling men aged 70–89 years and a median follow-up time of 5.90 years [28]. The patients were divided in quartiles according to baseline TSH and FT4. Men who developed dementia had higher baseline FT4 (16.5 ± 2.2 versus 15.9 ± 2.2 pmol/L, *P* = 0.004) but similar TSH (2.2 ± 1.4 versus 2.3 ± 1.6 mU/L, *P* = 0.23) compared with men who did not receive this diagnosis. Higher FT4 predicted new-onset dementia (11% increased risk per 1 pmol/L increase in FT4, *P* = 0.005; Quartiles Q2–4 versus Q1: adjusted HR = 1.76, 95% CI = 1.03–3.00, *P* = 0.04). There was no significant trend for quartiles of TSH or for TSH as a continuous variable against the outcome of new-onset dementia.

A negative association was shown in a longitudinal study with 660 subjects aged 73.3 ± 6.0 years and a follow-up of time of 3.8 ± 0.7 year [29]. In this Italian elderly cohort, increased baseline serum TSH did not predict risk of developing MCI or AD but association with an increased risk of VAD (vascular dementia) independent of sociodemographic and vascular risk factors. The patients were divided in tertiles according to baseline TSH. The highest TSH tertile had a threefold higher increased risk of VAD (OR: 3.25, 95% CI: 1.01–10.77, *P* = 0.048) compared to the lowest tertile. Adjusted risk of VAD increased about 60% for each 1-SD increase of log-transformed TSH (OR: 1.61, 95% CI: 1.06–2.44, *P* = 0.025). No association was found between serum FT4 considered as a continuous variable and risk of MCI and dementia. 

In our prospective study, a subsample of 200 nondemented subjects taken from the Kungsholmen project, a population-based study among people ≥75 years, and a mean follow-up time of 6.7 years, we found no association between TSH as a continuous variable (OR: 0.95, 95% CI (0.73–1.25, *P* = 0.75) and the development of AD after adjustment with age, gender, and education [30]. Neither did a classification in tertiles show a connection with development of AD. There was no correlation between TSH and age (*r* = −0.084, *P* = 0.239) or TSH and MMSE (*r* = −0.046, *P* = 0.552).

*Cross-Sectional/Case-Control Studies (Table 2)*. From the Sao Paulo Ageing and Health Study with 2 072 participants, a cross-sectional study with 1 119 community-dwelling persons of 65 years and older was made. 1 086 of those presented normal thyroid function and 33 SH [31]. After adjustment for age and using people with normal thyroid function as reference group, an association between SH and any type of dementia and VAD was found (OR 4.1; 95% CI, 1.3–13.1) and (OR 5.3; 95% CI, 1.1–26.4), respectively. No association was found between SH and AD. The results were confirmed with TSH presented in quintiles. The lowest serum TSH quintile showed a more than 3-fold increase in risk for all types of dementia (OR 3.6; 95% CI, 1.4–8.9) and for VAD (OR 9.3; 95% CI, 1.1–75.3) after multivariate adjustment compared to the third quintile. Analyzing data according to gender showed an association of SH with dementia and AD for men (OR 8.0; 95% CI, 1.5–43.4) and (OR 12.4; 95% CI, 1.2–128.4, resp.). No association was found between women with SH with any type of dementia or VAD in the sample.

 Also, the population-based CHIANTI study with 916 subjects aged 65 years and older found an independent association between SH and cognitive impairment [32]. The study of the relationship between thyroid function and cognitive performance was focused on older participants affected by SH, the most prevalent thyroid function abnormality in the study population (*n* = 71). In euthyroid participants, TSH and FT3 declined with age, whereas FT4 increased. In adjusted multivariate regression analysis the likelihood of having cognitive impairment associated with SH versus euthyroidism was more than a 2-fold risk of scoring less than 24 out of 30 on the MMSE (HR = 2.26; 95% CI, 1.32–3.91; *P* = 0.003).

In a pilot study, forty euthyroid AD patients aged 67.08 ± 6.92 were evaluated on their thyroid status (TSH, TT4 and TT3), cognition (MMSE), neuropsychiatric symptoms (Neuropsychiatric Inventory (NIP)), and depression (Hamilton Rating Scale for Depression (HAMD_17_)) [33]. Multivariate linear regression analyses were conducted to examine the relationships between thyroid hormone concentrations, demographics, and cognitive and mood measurements. Core score of HAMD was significantly associated with the serum level of TSH (*β* = 0.395, *P* < 0.01). No significant association was found between thyroid hormone levels and cognition, but patients with symptoms of agitation and irritability had lower serum TSH compared to those without these symptoms (*t* = −2.130, *P* = <0.05; *t* = −2.657, *P* = <0.05) in independent samples *t*-test.

A cross-sectional study involving 1219 community-dwelling subjects was taken from the Longitudinal Aging Study Amsterdam (LASA) [34]. Mean age of the participants was 75.5 ± 6.6 years. Global cognitive impairment and depression symptoms according to thyroid categories were not related to persons with SH (*n* = 34) and subclinical hypothyroidism (*n* = 64). 

Forty-three MCI cases and twenty-six healthy controls (mean age 61.1 ± 5.4 and 66.5 ± 6.2, resp.) participating in the Gothenburg MCI study comprised the sample for the study by Quinlan et al. [35]. Among those with MCI, TT3 levels were inversely associated with cognitive performance across all domains. After stratifying MCI cases according to TT3 levels, those with relatively high TT3 levels showed impairment in memory as well as in visuospatial and executive functions. TT3 was associated with a neuropsychological profile typical of prodromal AD among those with MCI. No association was found between TSH, TT4, and FT4 and dementia.

## 4. Discussion

 Eleven studies that met our criteria have examined the connection between SH and cognition. Six of these studies, including four with longitudinal analyses, have shown a consistent finding of an association of SH, or low serum TSH within the reference interval, with cognitive impairment or dementia. 

There are several explanations that can clarify these findings. TSH targets the thyroid gland and triggers the release of TH. TSH is released in response to TH-releasing hormone (TRH) from the hypothalamus. As the circulating concentrations of TH rise, the rates of TRH and TSH production decline. A low TSH level is found in people with hyperthyroidism and can also be a result of T4 substitution which leads to suppressed TSH in hypothyroids in a feed-back way. Some decrease in TSH secretion in older subjects has been supported by the observation that among patients with primary hypothyroidism, serum TSH concentrations are lower in older patients as compared with younger ones [36]. One explanation for the decrease in TRH and TSH secretion in older subjects could be their lesser need for T4 secretion. Hence, TSH secretion most likely decreases slightly with age, although the serum TSH concentrations generally remain within the reference range for younger subjects [37]. One theory is that the organic brain diseases causing cognitive impairment also reduce TRH secretion from hypothalamus and other brain areas. There are projections from many areas of the brain to the paraventricular nucleus containing the TRH-secreting neurons, such as the C1-3 adrenergic neurons of the brainstem and the hypothalamic arcuate nucleus which apply different effects on the hypophysiotropic TRH neurons [38]. Hence, TRH has a more widespread role as a central nervous system (CNS) transmitter, and the brain involution of the dementia process may lead to a widespread perturbation of neurotransmitters, together with TRH.

An alternative explanation might be that mild endogenous SH reflects true thyroid overactivity causing extreme thyroid hormone action on the CNS. Against this theory is the fact that only 20% of individuals with SH have a suppressed TSH. The low but not suppressed TSH in the 0.1–0.4 mU/L range is most commonly seen as a expression of nonthyroidal infirmity and is associated with lowering circulating FT3, rather than thyroid hormone excess. 

If this explanation would be true, individuals with the lowest TSH were to show the most cognitive impairments. This was not shown in the studies of Forti et al. where the subjects in the highest tertile of TSH had a threefold higher increased risk of VAD and Vadiveloo et al. with the most marked cognitive effects in grade I SH group [25, 29]. However, data on the influence of FT3 and FT4 levels on the development or worsening of dementia is conflicting [9, 39].

As the thyroid metabolism changes with age, low serum TSH and elevated FT4 levels may be markers of biological age reflecting reduced clearance capacity of thyroid hormones. The epidemiological studies in our review identify a group of subjects within a cohort who have more advanced biological age, which includes cognitive impairment and dementia. Anyhow, de Jong et al. found a 20% increased risk for dementia and a 30% risk for AD for each standard increase in serum FT4 in the Honolulu Heart Program [26]. Individuals with cognitive impairment and dementia often have a high burden of comorbidity that can lead to reduced serum TSH. A lot of medications for conditions other than thyroid diseases can also reduce the concentrations of serum TSH [40]. It is possible that this explanation contributes to some of the association between SH and dementia. 

In this focused review, we have performed an examination between low serum TSH and cognitive impairment in older people. In general, taking into account the largest and most powerfully designed studies, there is a strong body of evidence supporting the association between SH and cognitive impairment.

The scarce number of publications on these topics indicates the need of more research especially regarding longitudinal and interventional studies, thus hopefully robustly enabling confirmation or rejection of causality between TSH abnormalities and dementia.

## Figures and Tables

**Table 1:** Longitudinal population-based studies. Relationship between subclinical hyperthyroidism (SH) and cognitive decline.

Author	Study size (*n*)	Mean age	Followup (years)	Participants thyroid status	Thyroid function (normal range)	Outcomes
Vadiveloo et al., 2011 [25]	12.115	66.5 ± 15.9	5.6 (median)	SH and euthyroid	TSH (0.4–4.0 mU/L) FT4 (10–25 pmol/L) FT3 (0.9–2.6 nmol/L)	Positive association SH and dementia

de Jong et al., 2009 [26]	615	77.5	5	SH and euthyroid	TSH (0.4–4.3 mU/L) FT4 (0.85–1.94 ng/L)	Positive association FT4 and dementia/AD

Tan et al., 2008 [27]	1.864	71	12.7	SH and euthyroid	TSH (0.5–5.0 mU/L)	Positive association Lowest and highest tertile TSH and AD

Yeap et al., 2012 [28]	3401	76.8	5.9	Euthyroid only	TSH (0.4–4.3 mU/L) FT4 (10–23 pmol/L)	Positive association FT4 and dementia

Forti et al., 2012 [29]	660	73.3 ± 6.0	3.8 ± 0.7	Euthyroid and subclinical hypothyroidism	TSH (0.45–4.5 mU/L) FT4 (10.3–25.7 pmol/L)	No association TSH and MCI/AD Positive association High TSH and VAD

S. Annerbo et al., 2009 [30]	200	81.0 ± 4.6	6.7	All thyroid status (mean TSH 1.76 ± 1.03)	TSH (0.2–4.0 mU/L) TT4	No association TSH and AD

**Table 2:** Cross-sectional studies. Relationship between subclinical hyperthyroidism (SH) and cognitive decline.

Author	Study size (*n*)	Mean age	Participants thyroid status	Thyroid function (normal range)	Outcomes
Bensenor et al., [31] 2010	1119	*D* = 78.5 ND = 71.9	SH and euthyroid	TSH (0.4–4.0 mU/L) FT4 (0.77–2.19 ng/L)	Positive association SH and dementia especially vascular dementia

Ceresini et al., [32] 2009	916	>65	SH and euthyroid	TSH (0.46–4.68 mU/L) FT4 (0.77–2.19 ng/L)	SH group had significantly lower MMSE compared to the euthyroid group

Zhang et al., [33] 2012	40	67.08	All thyroid status	TSH (0.3–5.0 mU/L) TT4 (58.1–140.6 nmol/L) TT3 (0.92–2.97 nmol/L)	Lower TSH in euthyroid AD patients with agitation and irritability symptoms

de Jongh et al., [34] 2011	1219 (34 SH)	75.5	Euthyroid, SH, and subclinical hypothyroidism	TSH (0.3–4.5 mU/L) FT4, FT3	No association SH and impaired global cognitive function

Quinlan et al., [35] 2010	69	60.9–66.8	All thyroid status	TSH, FT4, TT4, TT3 (1.4–1.6 nmol/L)	Higher TT3 was associated with more cognitive impairment in MCI group
